# Experiences of People With Poorly Controlled Type 2 Diabetes Using Telemonitoring: Qualitative Study Embedded in a Feasibility Trial

**DOI:** 10.2196/89964

**Published:** 2026-05-28

**Authors:** Iben Engelbrecht Giese, Sisse Heiden Laursen, Pernille Frøstrup Barington, Peter Vestergaard, Stine Hangaard

**Affiliations:** 1 Department of Health Science and Technology, Faculty of Medicine, Aalborg University, Selma Lagerløfs Vej 249, Gistrup, North Denmark, 9260, Denmark, 45 99409940; 2 Steno Diabetes Center North Denmark, Aalborg University Hospital, Gistrup, North Denmark, Denmark; 3 Clinical Nursing Research Unit, Department of Clinical Medicine, Aalborg University Hospital, Aalborg, North Denmark, Denmark; 4 Department of Endocrinology, Aalborg University Hospital, Gistrup, North Denmark, Denmark

**Keywords:** type 2 diabetes, telemonitoring, feasibility trial, qualitative research, telehealth, self-management, patient education

## Abstract

**Background:**

Telemonitoring has been shown to improve glycemic control in type 2 diabetes, but the optimal design for effectively integrating self-management education remains unclear. Including patient feedback in the design process can enhance usability, increase engagement, and improve the feasibility and effectiveness of the intervention in real-world settings.

**Objective:**

This study aims to explore participants’ experiences and the acceptability of 2 different telemonitoring intervention designs and trial procedures used in a feasibility trial among people with non–insulin-dependent type 2 diabetes.

**Methods:**

Using a qualitative research design, semistructured interviews were conducted with participants who had completed the telemonitoring intervention. The interviews were analyzed using the thematic approach outlined by Braun and Clarke.

**Results:**

A total of 12 participants were interviewed. Four major themes emerged from the analysis: (1) acceptance of and experience with telemonitoring and devices, (2) structure and flow of the intervention, (3) relationship with and support from health care professionals, and (4) learning to live with diabetes. Participants found the devices easy to use, particularly self-monitoring of blood glucose, which was perceived as highly relevant and informative. Technical challenges were primarily related to the activity tracker and initial device setup. The measurement schedule supported self-management, though some participants found it inflexible and difficult to integrate into daily life. Continuous communication with health care professionals was highly valued and fostered trust. Participants reported increased insight into the relationship between lifestyle behaviors and blood glucose levels, which motivated healthier dietary choices and increased physical activity. Participants described that telemonitoring enhanced their understanding of diabetes and supported their engagement in self-management, although preferences for measurement types and frequency varied.

**Conclusions:**

Participants reported overall satisfaction, attributing it to structured monitoring and consultations with health care professionals that supported self-management. Blood glucose, physical activity, and diet were considered the most relevant data types. Tailoring the intervention to user priorities and improving the usability of devices and the intervention structure may increase engagement and motivation. Integrating continuous glucose monitoring may further reduce the burden associated with self-monitoring in future telemonitoring interventions for people with type 2 diabetes.

## Introduction

Effective management of type 2 diabetes (T2D) mellitus is critical, as maintaining optimal glycemic control is vital to reducing the risk of diabetes-related complications, such as retinopathy, neuropathy, and cardiovascular diseases [1,2]. However, achieving optimal glycemic control is challenging due to the complexity of the disease, and people with T2D are required to take a significantly active role in managing their diabetes outside of hospital settings. This self-management includes performing complex care activities, making lifestyle adjustments, and sustaining adherence to prescribed medication [3-5].

Telemedicine has emerged as a promising approach to support people with T2D in improving glycemic control and achieving treatment goals [6-11]. Telemedicine includes exchanging information or data and delivering personalized remote feedback between health care professionals (HCPs) and patients [12,13]. The effect of telemedicine on diabetes management is inconclusive, yet there is a positive trend in terms of glycemic control [7,14-17]. A systematic review and meta-analysis by Faruque et al [14] demonstrated improved glycated hemoglobin A_1c_ (HbA_1c_) levels in people with T2D who used telemedicine as an addition to standard care. A systematic review, meta-analysis, and meta-regression by Hangaard et al [18] concluded that telemedicine, particularly when including a telemonitoring component, can be a valuable supplement to usual care, especially for patients with poor glycemic control. Furthermore, incorporating self-management education into telemonitoring may strengthen patients’ ability to manage daily care and mitigate diabetes complications [19,20].

Skills, knowledge, and ability are essential components in diabetes self-management education to ensure effective daily diabetes care [21]. Including an educational self-management component in a telemedicine solution may have the potential to provide a foundation for people with T2D to navigate care activities, potentially helping to control disease progression and reduce the risk of diabetes-related complications [3,19,20,22]. However, despite these promising benefits, the optimal design of a telemonitoring solution that effectively integrates self-management education for people with T2D remains unclear.

When designing a telemonitoring intervention, it is crucial to gather information about participants’ experiences with the design. Understanding patient perspectives can provide valuable insights into practicality, usability, and overall acceptability [23]. It also helps identify barriers and facilitators to engagement, ensuring the telemonitoring intervention is tailored to patient needs and thereby more likely to be successfully implemented in a real-world setting [23-25]. The aim of this study is to explore participants’ experiences and the acceptability of 2 different telemonitoring intervention designs and trial procedures used in a feasibility trial among people with T2D.

## Methods

The study was conducted and reported according to the COREQ (Consolidated Criteria for Reporting Qualitative Research) checklist (Checklist 1) [26].

### Setting and Study Design

This study was conducted in the Region of North Jutland, Denmark, and was a part of a telemonitoring feasibility trial. The trial protocol has been published elsewhere [27].

This qualitative study used a hermeneutic phenomenological approach to explore participants’ lived experiences and perceptions of intervention acceptability. This theoretical framework was chosen due to its capacity to support an in-depth, interpretive understanding of how individuals make sense of their experiences within the specific context of telemonitoring [28] and informed both data collection and the analytical process.

### Intervention Context

**Figure 1.**
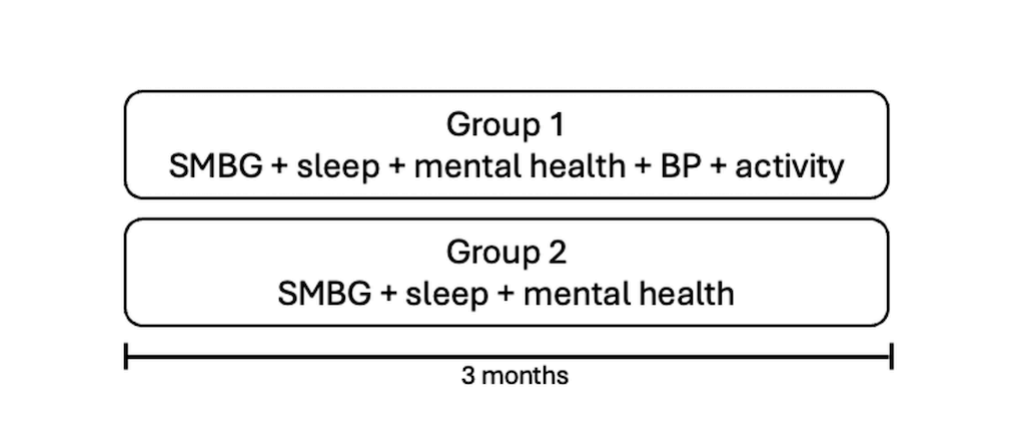
Overview of the 2 intervention designs. BP: blood pressure; SMBG: self-monitoring blood glucose.

Two telemonitoring intervention designs were evaluated, with the aim of identifying the most suitable design for a forthcoming large-scale randomized controlled trial. These 2 designs, based on essential components of effective T2D management [29-35], were tested over a 3-month period (Figure 1). Participants from groups 1 and 2 measured self-monitoring blood glucose (SMBG; Contour Next One), sleep, and mental health. Group 1 additionally monitored blood pressure (BP; A&D UA-651BLE) and step counts (Beurer AS97).

Participants with non–insulin-dependent T2D were recruited through general practices in 4 municipalities in the Region of North Jutland, Denmark, as part of the telemonitoring feasibility trial. In the Danish health care system, general practitioners serve as patients’ primary point of contact and act as gatekeepers to specialized care. The participating general practices identified and referred eligible patients during routine diabetes-related consultations. Adults aged 18 years or older with poorly controlled T2D (HbA_1c_ >58 mmol/mol), diagnosed for at least 12 months, who could read and understand Danish and were willing to use a smartphone were eligible for participation. Prior to the intervention start, participants received an introductory consultation with their assigned municipal HCP, including an intervention overview and device training. All municipality HCPs had a nursing background with a key focus on diabetes management in a municipality health care setting.

Participants followed a structured monitoring plan. During the first 6 weeks, they measured on 2 weekdays per week and submitted data to HCPs via the tablet. The remaining 6 weeks were tailored with HCPs, typically involving 2 to 3 measurement days per week. Biweekly scheduled consultations with HCPs supported data review of self-monitored data and diabetes management discussions. Participants could log dietary intake on measurement days to further support these conversations.

Participants were asked to follow a blood sampling schedule with HbA_1c_ measurements prior to the intervention, after the 3-month monitoring period, and again at 6 and 12 months post initiation. Furthermore, four questionnaires were completed prior to and at the end of the intervention: the World Health Organization Five Well-being Index, the Problem Areas in Diabetes Questionnaire, the Short Form 12 Questionnaire, and the Patient Activation Measure questionnaire, comprising 35 questions in total.

### Participants and Recruitment

Participants who completed the telemonitoring trial were eligible for interview and were recruited continuously by one author (IEG) from May 2024 to January 2025. At trial completion, HCPs asked participants if they were interested in an interview; those who agreed had their contact details forwarded to IEG, who then contacted them by phone.

### Researcher Characteristics and Reflexivity

One author (IEG) conducted all interviews. IEG has a background in physiotherapy and holds a master’s degree in clinical science and technology. At the time of the study, IEG was employed as a research assistant working within the field of diabetes and telemonitoring. IEG had prior experience in the qualitative research field, particularly with semistructured interviews. There was no personal relationship between IEG and the participants.

### Data Collection

The data were collected through semistructured interviews. The interview guide was developed by the authors IEG and SH, and was based on the medical technology assessment model [36], focusing on 3 domains: technology, patient, and organizational aspects. Within each domain, open-ended and follow-up questions were formulated to explore participants’ experiences with and acceptance of the 2 designs. A pilot test of the interview guide with one coauthor, SHL, who was not involved in its development, led to minor wording and sequencing adjustments before finalization.

Interviews were conducted once, either in the participant’s home or at their respective health center, with only the participant and interviewer present. Interviews lasted between 28 and 44 minutes. A Dictaphone was used to record and transcribe verbatim using Microsoft Word. Audio recordings and transcripts were stored on a protected server at Aalborg University. Transcripts were pseudonymized, and participants were referred to as “Id 1,” “Id 2,” and so forth. To enhance accuracy and credibility, participant validation was performed, allowing them to review and comment on their transcripts. The final transcripts were subsequently uploaded to NVivo 15 (version 15.1.1; Lumivero) for analysis.

### Data Analysis

Data analysis followed the 6-phase reflexive thematic approach outlined by Braun and Clarke [37,38]: (1) familiarization with the data, (2) generating initial codes, (3) searching for themes, (4) reviewing themes, (5) defining and naming themes, and (6) producing the final report [37]. IEG transcribed the interviews, after which IEG, SHL, and SH read them repeatedly for familiarization. They independently generated initial codes. These codes were discussed to reach a shared agreement on how to apply them consistently across the dataset. Agreed-upon codes were organized by the same 3 authors into potential candidate themes and subthemes and afterward reviewed and refined to ensure they accurately reflected the data. Finally, themes were further refined, named, and finalized for reporting.

### Ethical Considerations

The telemonitoring feasibility trial was approved by the North Denmark Region Committee on Health Research Ethics (ID: N-20230026). The approved protocol included the assessment of participants’ experiences and the acceptability of the telemonitoring intervention designs as study outcomes. Participants received written information and were informed of their right to a 24-hour reflection period before consenting to participate. All participants provided both written and verbal informed consent to participate in the study, to be audio-recorded, and to allow the use of their data for research purposes. All data were pseudonymized as described in the *Data Collection* section or presented in aggregated form only to prevent identification of individual participants, thereby ensuring privacy and confidentiality. Participants did not receive any compensation for their participation.

## Results

### Participants Characteristics

A total of 12 participants were invited to participate in an interview, all of whom accepted. Participants had a mean age of 63.8 (SD 9.6) years and a mean diabetes duration of 8.2 (SD 6.5) years. Two participants were female, and all participants were of Danish origin. Five participants had primary school or high school as their highest level of education, while the remaining participants had completed vocational education or higher education. Five participants belonged to group 1, while 7 belonged to group 2.

### Interview Findings

**Figure 2.**
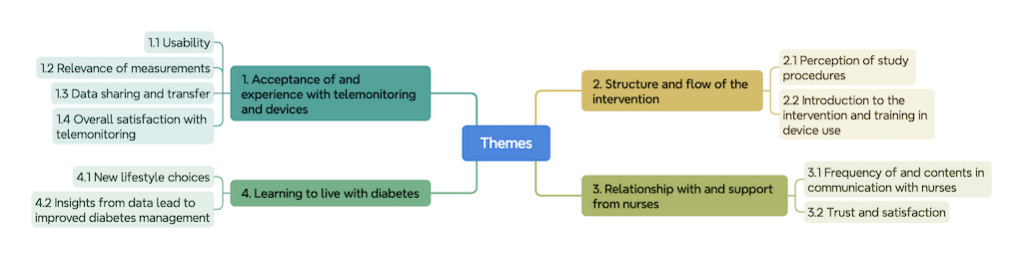
Overview of the themes and related subthemes.

Four themes emerged from the analysis. Each theme included 2 to 3 subthemes (Figure 2). A detailed overview of the themes, subthemes, and associated codes is available in Multimedia Appendix 1.

#### Theme 1: Acceptance of and Experience With Telemonitoring and Devices

This theme captures participants’ initial attitudes toward the technology and their experiences using the devices in everyday life.

##### Subtheme 1.1: Usability

The SMBG device was perceived as easy to use, and all participants were able to perform measurements independently:

*But it [SMBG device] worked perfectly. If I can figure it out, then anyone can. [...] It wasn’t a problem at all. It was very intuitive and child-friendly.*

[Id 2]

A few participants experienced minor technical issues, primarily related to device setup. Once these issues were resolved, no further problems occurred:

*My blood glucose device, it wasn’t properly set with the year, so I missed some data in the beginning […] But then it just worked fine.*

[Id 1]

None reported any issues with the user-friendliness or technical functionality of the BP device. All participants were able to perform the measurement themselves, with the exception of 1 participant who received support from a partner:

*She measured my BP. […] To check that it was all fine. So I did that to make sure it was as it should be. But otherwise I did the other measurements myself.*

[Id 1]

Several participants experienced technical issues with the activity tracker, leading some to stop using it or switch to personal devices. One participant also reported discomfort from the strap:

*It [activity tracker] wouldn’t send—completely dead—even though it was set up correctly, and then nothing was recorded. And there was one more thing ... If you’re working, that stiff plastic strap really digs into your skin. It just doesn’t work.*

[Id 5]

The tablet and associated telemonitoring system were generally reported as intuitive and easy to use. Several participants expressed satisfaction with the system’s functionalities, particularly the ability to send messages to HCPs and upload pictures of their food, which provided an easy and time-saving way to communicate with HCPs:

*I mean, if you had to call each other all the time, it would be completely hopeless. You can just write and then you get a reply quickly. […] Yes, communication on the system works.*

[Id 5]

Participants used the system’s overview function differently. Some valued it for tracking glucose trends, while others focused on single readings. Participants suggested clearer visualizations and the addition of features such as step counts and dietary logs.

*And I probably would have preferred that the dietary and drink log, that it existed in a digital format.*

[Id 4]

Only a few participants involved a partner. They primarily assisted participants in performing measurements correctly or provided support in using the system on the tablet:

*It’s my wife who managed the tablet. That was a condition for me to participate, because I’m not that strong with computers.*

[Id 5]

##### Subtheme 1.2: Relevance of Measurements

Only participants who discovered hypertension during the intervention found BP measurements relevant. The other participants found BP measurements easy to perform but did not perceive them as relevant. Several participants considered activity tracking important and meaningful:

*That thing about my steps, I’ve actually found it important, that there is some level of activity [to manage the diabetes].*

[Id 4]

Measuring blood glucose was considered essential by all participants, as it provided insights into how various factors affected these levels and how to respond appropriately before complications arose. For many, blood glucose monitoring was one of the most valuable aspects of the intervention:

*Well, it’s the blood glucose monitoring [that’s been the best part]. That’s definitely the one that has given me something to think about.*

[Id 12]

Most participants reported no discomfort related to SMBG finger pricks, though a few found it painful and uncomfortable due to calloused skin or spasticity, requiring repeated use of the same fingers. While acknowledging the relevance of measuring blood glucose, these participants expressed a preference to use alternative methods:

*Well, of course I’d prefer to do without it. Like I said, if there were something that could measure it electronically, then you’d avoid all those finger pricks and the time you have to spend pricking yourself.*

[Id 1]

Several participants expressed interest in using a continuous glucose monitor (CGM), highlighting advantages such as continuous glucose tracking, easier data access, no need to remember the timing of measurements, and not having to carry SMBG equipment. A few participants, however, found CGM unnecessary due to a perceived excess of measurements and a preference for occasional spot checks:

*So if you just check it at regular intervals, that should be enough. And if it really goes completely wrong, you can always take some more tests.*

[Id 11]

Several participants perceived the questions concerning sleep and mental health as lacking relevance to their diabetes management, noting that their responses often reflected external factors rather than changes related to T2D.

*There are everyday things that will influence some of the measurements. Like, my knee affected my sleep because it hurt so much, and I could wake up from it—so it wasn’t necessarily because of my diabetes. If my knee hurts for a longer period, it affects my mood. Then I might be more irritable. So, there are certain factors that will influence some of the data.*

[Id 9]

Conversely, other participants considered the frequency and scope appropriate and suggested expanding them to capture more nuanced aspects of sleep and mental health, thereby increasing their perceived applicability.

Experiences with the dietary log varied, with some choosing not to use it due to the effort involved, while others used it throughout the intervention period, finding it helpful in identifying links between blood glucose spikes and dietary intake.

*You can relate it to what you’ve eaten, because now I can see that after breakfast, it [blood glucose] goes up. So then I think—what did I eat for breakfast? And that helps me understand it better*.

[Id 4]

##### Subtheme 1.3: Overall Satisfaction With Telemonitoring

Several participants expressed satisfaction with participating in the intervention. It provided them with support and valuable knowledge about their T2D and served as a motivational push toward lifestyle changes. The intervention encouraged reflection on their personal situation and emphasized the importance of taking their condition seriously:

*I think it has been an interesting experience to be a part of. I’ve never really paid attention to my diabetes, and now I’ve realized that you need to take care of it.*

[Id 12]

A few participants noted that receiving the intervention closer to the time of their T2D diagnosis would have been preferable. They believed that earlier intervention could enhance their understanding of factors influencing blood glucose levels and, consequently, support better glycemic control:

*If I had had this [the intervention] back in 2022, where I could go in and measure, maybe I would have made more progress and gotten my numbers [blood glucose levels] down faster.*

[Id 4]

### Theme 2: Structure and Flow of the Intervention

This theme focuses on participants’ experiences with the structure and flow of the intervention, including procedures, monitoring, and training.

#### Subtheme 2.1: Perceptions of Study Procedures

Participants’ experiences with the monitoring plan varied. For some, the plan was straightforward and provided clear guidance, helping them stay organized with their measurements:

*Yes, I also thought it was [easy to follow the monitoring plan]. [...] I would mark it with a cross when I had completed a week and then moved on to the next, so I could remember it.*

[Id 8]

Others encountered challenges with timing and integrating measurements into daily life. Social activities, work, and everyday routines often interfered with scheduled time points for blood glucose measurements:

*I can’t remember—was it 1.5 to 2 hours after eating? It could easily shift. Sometimes I had to do it earlier because I knew I wouldn’t be able to later. [...] In weekends, it was like—well, we had been out in the garden, and then I’d go oh no, what time is it? I need to go measure my blood sugar. It was a bit difficult sometimes.*

[Id 7]

Some participants forgot to take measurements and expressed a need for a more flexible plan that could accommodate differences between weekdays and weekends. While several appreciated the structure, others found the measurement frequency excessive.

In some cases, participants had to bring devices to work or social events, which led to practical inconveniences and feelings of discomfort:

*Right at the beginning, we had a few birthdays and parties to attend, and some of the days overlapped with the times I was supposed to measure my blood sugar. [...] It was the inconvenience of being dependent on it when sitting at a party. And at the places we went to, there weren’t any private restrooms—you had to stand by the sink, where everyone else would come in. I found that really unpleasant.*

[Id 7]

While none of the participants considered the blood sampling schedule (HbA_1c_ at baseline, 3 months, 6 months, and 12 months) itself to be a challenge, they did note a lack of flexibility in scheduling, as blood samples in primary care could only be ordered up to 4 months in advance:

*I’ve booked appointment for one in three months. But for the one in six months, I couldn’t get an appointment—their system couldn’t schedule that far ahead.*

[Id 11]

#### Subtheme 2.2: Introduction to the Intervention and Training in Device Use

Several participants emphasized the relevance of the introductory consultation with HCPs, where they were further introduced to the trial and its procedures:

*It was actually quite nice to hear about it, because I didn’t really know what I was being offered. It was a surprise, but it was also good to hear what I was getting into, instead of just being handed something.*

[Id 1]

Following the first consultation, HCPs provided participants with a training session on how to use the devices. Several participants expressed satisfaction with the training and found it easy to learn.

### Theme 3: Health Care Professional Relation and Support

This theme examines how communication with HCPs influences participants’ experience, highlighting the role of support and trust in fostering motivation and acceptability.

#### Subtheme 3.1: Frequency and Content of Communication With Health Care Professionals

Several participants were satisfied with the biweekly scheduled conversations with their HCPs. Some also appreciated being able to contact their HCPs via the tablet outside of these scheduled sessions:

*No, it is fine, because I have been able to write to her if there was anything. And sent what I thought could be relevant regarding food and such. Well, I think it fits quite well.*

[Id 4]

In contrast, some preferred individualized plans, with consultation frequency adjusted based on their data and HCPs’ professional judgment. Most participants had contact with their HCPs outside scheduled consultations, primarily via the tablet’s messaging function. This communication often involved minor technical issues or practical matters, such as scheduling the next consultation. This was highly valued as it provided quick resolutions to minor problems. Biweekly consultations mainly focused on self-management, emphasizing how blood glucose levels were influenced by diet, physical activity, sleep, and medication:

*It has worked fine. Really well. During those bi-weekly conversations, we have delved into the [blood glucose] values, and now you can see why something was off there, well, it was because I was at a birthday party or something like that.*

[Id 1]

#### Subtheme 3.2: Trust and Satisfaction

Participants were highly satisfied with their consultations with HCPs, valuing the relevance of the topics and HCPs’ ability to communicate clearly and provide support. A few participants expressed a desire for in-depth explanations of the physiological mechanisms behind their diabetes. Some specifically mentioned feeling reassured that someone was keeping an eye on their data:

*I knew that someone was keeping an eye on me and would help me get a clear direction if I had any values that were completely off […]. So, it has actually been a help for me.*

[Id 1]

### Theme 4: Learning to Live With Diabetes

This theme highlights how the intervention enhanced participants’ insight into diet, activity, and blood glucose, motivating healthier choices and supporting self-management.

#### Subtheme 4.1: New Lifestyle Choices

Several participants reported an enhanced understanding of how diet and exercise influence blood glucose. They discovered that certain drinks and food, which they initially believed to be beneficial, caused an increase in blood glucose levels:

*Yes, I do think a bit more about it [diet]. [...] I can see from the data that what I eat has an impact. There are certain things where it’s clear that I should avoid them—or at least limit them.*

[Id 10]

Several participants expressed how increased exercise levels were visible in the blood glucose levels, and how they gained insight into how exercise can be used to reduce high blood glucose levels.

*But what I’ve probably learned from it [the intervention] is that when winter comes and we can’t get out and be active in the evenings, then we need to find something to do in the evening. […] I mean, we used to go for walks by the sea in the evenings, and you could see that is when it [blood glucose] dropped. And it kept doing that.*

[Id 6]

Increased insight into the effect of diet and exercise led several participants to make lifestyle changes, including healthier choices in their everyday diet based on their SMBG, and more physical activity:

*Yes, we talked a lot about how my blood sugar was way too high. So, I needed to bring it down. I had to start getting some exercise. So now I go for a walk every day. [...] And I drink a lot of water now. That’s something I’ve started. I used to drink a lot of soda.*

[Id 12]

A few participants did not make changes, feeling content with their current habits and viewing their diabetes as well controlled, thus seeing no reason to adjust:

*I’ve never had any problems. If I did have problems, I think I’d be more willing to make changes if something could help. But I’ve never felt like I have diabetes. [...] We don’t really change anything.*

[Id 6]

#### Subtheme 4.2: Insights From Data Improved Diabetes Management

Monitoring their data enabled participants to gain a deeper understanding of their T2D. Many reported that tracking fluctuations in blood glucose levels increased their awareness of how various factors influenced their condition. Some compared daily measurements, helping them recognize patterns and changes over time.

*Well, I could see those dots marked at specific times and dates, showing how high the levels were. Over time, it was actually quite interesting to observe the patterns. [...] And if I looked in my food diary, I could usually find an explanation. In that way, I think it’s been very educational—you could see, “ah, there’s a reason for that.” It was easy to pinpoint.*

[Id 3]

Several participants experienced improved blood glucose levels during the intervention period, and some reported a lower HbA_1c_. These improvements were associated with enhanced well-being, including weight loss, reduced leg discomfort, and increased energy:

*But I remember there was a time when I had a lot of trouble with my legs. And I think it had something to do with the diabetes […]. They’ve definitely gotten better since I got it [the blood glucose level] down.*

[Id 8]

Some participants reported that the data helped them confirm that their current lifestyle behaviors were appropriate and that no further changes could be made to lower their blood glucose levels:

*It’s a confirmation that what I’m doing is the right thing. Because when you prick your finger and see that the numbers look good, then there must be something right about what you’re doing.*

[Id 11]

Some participants had their BP and blood glucose medication adjusted based on monitored data:

*I’ve just started on insulin now [right after the intervention], because apparently the other treatment didn’t work. So we’ll see if this does. I mean, I can’t increase my activity any further.*

[Id 5]

All participants reported an improved understanding of T2D. Weekly measurements and biweekly consultations with HCPs provided structure and enhanced knowledge of physiological mechanisms underlying blood glucose regulation and its responses to everyday activities. It served as a reminder of living with a disease that requires attention, even in the absence of symptoms. This increased sense of control enhanced self-management:

*Yes, I do think so [learned something about T2D]. All the different factors that actually play a role. Also, that even if you are very disciplined, you still need to take measurements to really stay in control. Otherwise, you’re not truly in control.*

[Id 5]

A few participants expressed that the data confirmed their existing knowledge about their T2D, as the data clearly illustrated how blood glucose levels responded to everyday activities:

*Yes, I already knew that before. But now it’s been documented, made completely tangible—but I did know it. There are no real secrets when it comes to diabetes, about what you should avoid and what you should do. It’s more about how it’s carried out in practice. [...] I’ve become much more aware of how it actually works. Not just in theory, but I’ve seen it for myself.*

[Id 3]

## Discussion

### Principal Findings

This study explored experiences and perceptions regarding the acceptability of 2 different telemonitoring interventions and associated trial procedures among people with non–insulin-dependent T2D. SMBG, physical activity, and dietary logging were highly valued, while BP, sleep, and mental health monitoring were perceived as less relevant. Gaining access to personal data, combined with HCP consultations, provided valuable insights and enhanced perceived self-management competencies.

### Comparison With Prior Work

Prior research has demonstrated that the quality of sleep and mental health status are associated with glycemic control and are therefore relevant factors to monitor and address in diabetes management [30,31,39,40]. Surprisingly, participants in this study perceived these data as difficult to relate to diabetes, as they found it difficult to interpret the results or see their relevance for diabetes management. This highlights the importance of collecting data in telemonitoring that is meaningful and understandable to users. Previous research shows that perceived usefulness and ease of interpretation strongly influence sustained health monitoring [41,42]. This suggests that telemonitoring interventions must consider not only clinical validity but also users’ subjective experience of relevance [41,42]. Furthermore, if mental health or sleep-related measures are to be included in future telemonitoring interventions, considerations regarding how these data are presented and how frequently they are collected may be important to enhance perceived relevance and usability.

Participants greatly valued the supportive role of HCPs. This is consistent with previous research showing that people with T2D regard empathy, inclusion, clear communication, and personalized guidance as essential for maintaining motivation and engagement in self-management [43,44]. Preserving and strengthening this supportive role should therefore be a central consideration in future telemonitoring trials.

Access to their own health data such as blood glucose levels enhanced participants’ understanding of diabetes and motivated them to make lifestyle adjustments. This perceived empowerment aligns with previous research highlighting that telemonitoring interventions promote lifestyle modifications and an increased sense of disease ownership among people with chronic conditions such as diabetes [45]. In similar studies, participants valued the increased visibility of their blood glucose fluctuations and used the data to inform their decisions related to diet and physical activity, helping them maintain glycemic control [46,47].

Participants described SMBG as uncomfortable, time-consuming, and difficult to integrate into daily life. This experience is consistent with the findings of Ong et al [48], who identified pain from finger pricks, inconvenience of the procedure, and the need to carry devices as key barriers to SMBG. Several participants in this study wished to replace SMBG with CGM, not only to overcome the aforementioned challenges but also due to the advantage of continuous and easily accessible glucose data. Previous research has demonstrated that CGM significantly reduces HbA_1c_ compared to SMBG in people with T2D [49,50]. Moreover, CGM use in people with T2D enhances self-care behaviors, strengthens self-efficacy, and improves diabetes-related knowledge [51]. Thus, integrating CGM into future telemonitoring interventions may increase feasibility and acceptability.

### Limitations

A limitation of this study is that only participants who completed the telemonitoring feasibility trial were invited to participate in the interviews. Consequently, perspectives from those who withdrew from the telemonitoring intervention were not captured. The findings may overrepresent positive experiences and higher acceptability of the intervention, potentially overlooking critical feedback. This limits the transferability of the findings to a broader population of people with T2D. Future research would benefit from actively including participants in qualitative evaluations who withdrew from the telemonitoring intervention to provide a more nuanced understanding of experiences and acceptability.

An additional limitation of this study is the relatively small sample size (n=12), which may limit the transferability of the findings beyond the specific study context. However, it can be assumed that data saturation was achieved, as no substantially new perspectives emerged during the later interviews, suggesting that the key themes were sufficiently explored [52]. However, caution is warranted, as a small sample may not fully capture the diversity of experiences among individuals with T2D, particularly across different contexts.

The qualitative design constitutes a strength of this study, as it enables an in-depth exploration of participants’ lived experience and an assessment of the acceptability of the telemonitoring intervention designs. The hermeneutic phenomenological approach was therefore considered to be well suited to capture the nuanced insights [37,53]. However, this approach also entails an interpretive analytical process, which may introduce subjectivity and potential researcher bias, as the findings are shaped through the researchers’ preunderstandings [53]. To enhance analytical rigor, the analysis was guided by a structured analytical method and involved ongoing discussions among multiple authors throughout the analytical process [37]. However, qualitative data analysis remains inherently interpretive, and alternative interpretations of the data are possible. The findings should therefore be understood within the context of this analytical framework and with consideration of the limited generalizability inherent to qualitative research.

In conclusion, 12 people with T2D who completed the feasibility trial were overall satisfied with telemonitoring, mainly due to structured measurement combined with biweekly consultations with HCPs. Blood glucose, physical activity, and diet monitoring were perceived as particularly relevant, whereas BP, sleep, and mental health were considered less useful. Tailoring interventions to user-perceived relevance, providing clear feedback, and integrating CGM may enhance motivation and adherence in future telemonitoring interventions.

## Supplementary material

10.2196/89964 Multimedia Appendix 1 Detailed overview of themes, subthemes, and associated codes.

10.2196/89964 Checklist 1 COREQ checklist.

## Abbreviations

BP: blood pressure
CGM: continuous glucose monitor
COREQ: Consolidated Criteria for Reporting Qualitative Research
HbA_1c_: hemoglobin A_1c_
HCP: health care professional
SMBG: self-monitoring of blood glucose
T2D: type 2 diabetes
