# Supplementary material for: Experiences of People With Poorly Controlled Type 2 Diabetes Using Telemonitoring: Qualitative Study Embedded in a Feasibility Trial
Source: JMIR Form Res. 2026 May 28;10:e89964. doi: 10.2196/89964 (PMC13218280; doi:10.2196/89964)
Supplement: Multimedia Appendix 1 [file formative-v10-e89964-s001.docx]

| 1. **Acceptance of and experience with telemonitoring and devices** | 1. Usability | 1. Competences to perform SMBG 2. Competences to perform blood pressure 3. Preference for easy accessibility to blood glucose measurements 4. Factors influencing the utilization of the iPad 5. Usability – app 6. Usability - SMBG 7. Usability – blood pressure measurement 8. Assistance from relatives 9. Technical experiences with activity tracker 10. Technical experiences with SMBG 11. Technical experiences with blood pressure device 12. The utilization of app functions 13. Insufficient knowledge regarding app functions 14. Challenges with iPad 15. Discomfort with activity tracker 16. Activity tracker is inaccurate in step counting 17. Satisfaction with bring your own device 18. Easy to understand visualization of data 19. Difficult to understand visualization of data 20. Concrete numbers are easier to understand than diagrams 21. Satisfaction with being able to communicate via the app 22. Suggestions for interface improvements 23. Suggestions for app changes |
| --- | --- | --- |
|  | 1. Relevance of measurements | 1. Satisfaction with the types of measurements 2. Relevance of SMBG 3. Relevance of blood pressure 4. Relevance of activity measurement 5. Preference for activity measurements 6. Preference for other types of measurements 7. Experience with SMBG 8. Experience with meal registration 9. Considerations regarding CGM 10. Experience with sleep registration 11. App-questions – flexibility 12. App-questions – relevance 13. App-questions – quantity 14. Other factors influencing responses to app-questions 15. Lack differentiation in activity levels |
|  | 1. Overall satisfaction with telemonitoring | 1. Overall satisfaction with telemonitoring 2. Preference for an earlier start |
| 1. **Structure and flow of the intervention** | 1. Perceptions of study procedures | 1. Questionnaire – flexibility 2. Questionnaire – relevance 3. Questionnaire – easy to answer 4. Measurement plan – flexibility 5. Measurement plan – easy 6. Measurement plan – difficult 7. Measurement frequency 8. Creating structure through measurements 9. Measurements can be difficult to remember 10. No contact with primary care during the intervention 11. Blood test in primary care 12. Lack of flexibility in blood test ordering 13. Adapt the intervention to everyday life 14. Adaptation to technology and measurements |
|  | 1. Introduction to the intervention and training in device use | 1. Clarifying conversation or nursing assessment 2. Distribution and setup of equipment 3. Positive experience with training in equipment 4. Information prior to consent |
| 1. **Relationship with and support from nurses** | 1. Frequency of and contents in communication with nurses | 1. Frequency of contact with nurses 2. Content of communication with nurses 3. Communication regarding data 4. Communication outside scheduled conversations |
|  | 2. Trust and satisfaction | 1. Support from nurses in communication with primary care 2. Trust in nurses 3. Satisfaction with conversations with nurses 4. Positive to be monitored 5. Need for more information about diabetes 6. Preference for support from other nurses |
| 1. **Learning to live with diabetes** | 1. New lifestyle choices | 1. Insight into the importance of diet 2. Insight into the importance of exercise 3. Changes in habits and behavior 4. The intervention doesn’t necessarily lead to changed behavior |
|  | 1. Insights from data lead to improved diabetes management | 1. Relate to your own data 2. Increased disease insights and control 3. The intervention leads to increased glycemic control 4. The intervention leads to increased overall well-being 5. The intervention confirms behavior 6. Assumption that learning fades over time 7. Medication adjustment during the intervention 8. Diabetes management prior to the project |
